# 4035 Astrocyte LDLR-Related Protein 1 Increases Cytokine Sensitivity – The Role of Glia in Recovery after Brain Damage

**DOI:** 10.1017/cts.2020.56

**Published:** 2020-07-29

**Authors:** Sadiya Ahmad, Pamela Reed, Shane Sprauge, Naomi Sayre

**Affiliations:** 1 University of Texas Health Science Center San Antonio

## Abstract

OBJECTIVES/GOALS: The limited treatment options for ischemic stroke patients have resulted in stroke being a leading cause of death and the primary cause of long-term disability in the U.S. Finding effective treatment options requires a better fundamental understanding of the ongoing processes that contribute to poor long-term outcome. METHODS/STUDY POPULATION: Expression of Apolipoprotein E4 predisposes stroke patients to poor long-term outcome. This study aims to test one possible mechanism by which ApoE4 contributes to cognitive decline after stroke. Here, we examine the effect of a major ApoE4 receptor, low-density lipoprotein receptor related protein 1 (LRP1) on sensitivity to stress in astrocytes. LRP1 binds and moves extracellular ligands and plasma membrane proteins into the endocytic system. Others have shown that LRP1 regulates cell-surface TNF receptor (TNFR1) in non-astrocytic cells. We propose That LRP1 similarly regulates TNFR1 in the central nervous system to attenuate inflammatory response after stroke. Studies have shown that ApoE4 slows the recycling of endocytic LDL receptors. We hypothesize that ApoE4 inhibits the ability of LRP1 to remove TNFR1 from the plasma membrane. This is expected to increase cytokine sensitivity, resulting in worse outcome after stroke. We investigated the effect of LRP1 on astrocyte TNFα signaling and response in immortalized ApoE null mouse astrocytes subjected to lentiviral-mediated knockdown of LRP1. The astrocyte response to TNFα stimulation was tested in a time dependent manner using Western blotting of NFkB pathway components, which are the downstream mediators of TNFα signaling. We also tested astrocyte viability after prolonged TNFα stimulation using Alamar Blue reagent. We found that LRP1 deficient cells have increased phosphorylation of NFkB upon TNFα stimulation, and that loss of LRP1 resulted in significant loss of astrocyte viability after prolonged stimulation. RESULTS/ANTICIPATED RESULTS: Altogether, our results indicate that loss of LRP1 renders astrocytes more sensitive to TNFα. Future experiments will focus on testing the influence of LRP1 on recovery after middle cerebral artery occlusion in mice. DISCUSSION/SIGNIFICANCE OF IMPACT: These studies will elucidate how astrocyte-LRP1 contributes to outcome after stroke, and helps us to understand one potential way that ApoE4 exerts pathological effects. A better understanding of the long-term processes after stroke will allow identification of therapies which improve the morbidity and mortality associated with stroke. CONFLICT OF INTEREST DESCRIPTION: NA.

